# Linking cardiological parameters and choroidal changes in patients with precapillary pulmonary hypertension: a cross-sectional study

**DOI:** 10.1038/s41598-025-17425-z

**Published:** 2025-08-28

**Authors:** Agnieszka Zonenberg, Katarzyna Ptaszyńska, Anna Szwedowicz, Emilia Sawicka-Śmiarowska, Remigiusz Kazimierczyk, Barbara Pieklarz, Izabela Zawadzka, Iwona Obuchowska, Aleksandra Leoniuk, Joanna Konopińska, Karol Kamiński, Diana A. Dmuchowska

**Affiliations:** 1 Ophthalmology Department, Medical University of Bialystok, 24a M.Sklodowskiej-Curie St., Białystok, 15-276 Poland; 2 Department of Cardiology and Internal Diseases, Medical University of Bialystok, Białystok, Poland

**Keywords:** Choroid, Choroidal thickness, Choroidal vascularity index, Pulmonary hypertension, Vascular bed, Sildenafil, Bosentan, Pathogenesis, Cardiology, Cardiovascular biology, Diagnostic markers, Diseases, Cardiovascular diseases, Eye diseases, Biomarkers, Diagnostic markers

## Abstract

Diagnosing precapillary pulmonary hypertension (PPH) is challenging, and prognosis remains poor. This study aims to explore the pathogenesis of choroidal changes in these patients and assess whether choroidal parameters could serve as noninvasive biomarkers reflecting cardiovascular status. This prospective, single-center study included 29 adult PPH patients and 37 healthy controls. One eye per patient was analyzed. All participants underwent a full ophthalmological examination, including spectral domain-optical coherence tomography, and a cardiological evaluation comprising medical history, physical examination, echocardiography, right heart catheterization, laboratory, and functional tests. Univariable regression analyses assessed associations between choroidal parameters and clinical, demographic, and ocular features. PPH patients showed significantly reduced choroidal thickness and volume in the ETDRS subfields compared to controls (*p* < 0.01 for all). However, luminal, stromal, and total choroidal areas, as well as the choroidal vascularity index, did not differ significantly between groups. No significant relationships were found between cardiological and choroidal parameters. The choroid is affected in PPH patients, but choroidal parameters do not appear to be reliable biomarkers for PPH evaluation. Further studies are needed to clarify the potential role of choroidal vascularity index in PPH.

## Introduction

Precapillary pulmonary hypertension (PPH) is a condition in which the increase in pulmonary arterial pressure is observed without postcapillary pressure increase. This group of pulmonary hypertension includes pulmonary arterial hypertension, that is a rare disease caused by progressive narrowing of the pulmonary arterial lumen, as well as chronic thromboembolic pulmonary hypertension caused by chronic occlusion of pulmonary arteries. Both types of PPH lead to a rise in pulmonary arterial pressure and pulmonary vascular resistance^1^. The symptoms of PPH may be influenced by the underlying or associated conditions that cause PH, as well as other comorbidities. In general, the prognosis is poor^2^. There are diagnostic and prognostic challenges and the cardiologic evaluation is time-consuming. It requires invasive techniques and experienced personnel to perform and interpret the results.

The pathogenesis of PPH involves various mechanisms, leading to changes in the pulmonary vasculature, right heart, systemic circulation, and other organs. One of the primary features is endothelial dysfunction, leading to the imbalance between vasoconstrictors (endothelin, thromboxanes) and vasodilators (nitric oxide, natriuretic hormones, prostacyclin). It leads to vascular remodeling and chronic hypoxia^3^. Furthermore, autonomic imbalance—marked by heightened sympathetic vasoconstriction and reduced parasympathetic dilation—exacerbates vascular tone dysregulation^4^. In addition to inflammation and thrombosis^5^, congestion also plays a role. As far as ophthalmology is concerned, in advanced stages, elevated pressures in pulmonary arteries may cause subsequent increase of blood pressure in superior vena cava, which in turn leads to an increase in eye venous pressure and overload of the venous vascular bed, among other things, such as dilated and tortuous epidural vessels^6^. One may assume an analogy in the choroidal vascular bed. A number of ocular manifestations have been reported in PPH, primarily in case reports and small case series^7,8^. This study is focused on choroidal abnormalities. Choroidal congestion and endothelial changes in patients with PPH may lead to multiple ocular complications, such as ciliary detachment, acute serous retinal detachment^9^, uveal effusion, that may cause temporary myopia and even periodic angle-closure glaucoma due to the forward displacement of the lens-iris diaphragm^7,8^.

The choroid is one of the most vascularized tissues in the human body. It functions to oxygenate the outer retina and remove metabolic waste products. Choroidal vasculature forms a major reserve of ocular hemodynamics as uvea can hold up to 85% of intraocular blood^10^. The choroid is predominantly under sympathetic control, with noradrenergic fibers mediating vasoconstriction to match systemic demands^11^. Lacking intrinsic autoregulation, choroidal flow varies directly with perfusion pressure, making it vulnerable to systemic venous congestion and pressure changes^12^. Parasympathetic-mediated vasodilation, and endothelial-derived factors—nitric oxide, endothelin and vascular endothelial growth factor —further modulate vessel tone and long-term vascular remodeling. This high-flow, low-resistance system with fenestrated capillaries remains highly sensitive to systemic hemodynamic and microvascular changes^11^.

The choroid is characterized by the following parameters: thickness, volume, and a relatively new indicator of its vascularization (choroidal vascularity index - CVI). Those data may be obtained by means of non-invasive, fast, and commercially available examination, the optical coherence tomography (OCT). The OCT is widely used in routine diagnosis and monitoring of various retinal and choroidal diseases. Choroidal thickness (CT) is dependent on many variables, including sex, age, blood pressure, time of the day, and axial length (AL) of the eyeball^13^. The CVI specifically analyzes the vascular component of the choroid, including all choroidal vessel layers, i.e. choriocapillaris, Sattler’s and Haller’s layer. The CVI is defined as the ratio of the luminal area (LA) to the total choroidal area (TCA). The CVI is a more stable parameter that could become a biomarker in the future, facilitating the diagnosis, monitoring, and prediction of the progression of systemic diseases^13–16^. The choroidal vasculature can be considered as ideal for the observation of generalized arteriolar and capillary pathology^17^. The pathomechanisms of PPH—namely autonomic dysregulation, elevated venous pressure resulting in venous overload and endothelial stress —are directly relevant to the choroid, which uniquely lacks autoregulatory capacity. Under these combined perturbations, non-invasive and widely available parameters such as OCT-derived choroidal thickness and the CVI may sensitively reflect the integrated hemodynamic and microvascular disturbances in PPH patients, serving as complementary biomarkers to repeated invasive assessments^11^. While choroidal thickness (CT) reflects overall tissue engorgement, CVI (by isolating the vascular lumen from the stromal tissue) directly quantifies choroidal vascular remodeling and congestion. This specificity renders CVI a more stable and sensitive biomarker for detecting subtle vascular alterations that may parallel hemodynamic changes in PPH .

The information in the literature regarding OCT-related morphological choroidal parameters in PPH patients, CVI in particular, is sparse and with the current study we would like to fill this gap.

The aim of the study is to get a better insight into the pathogenesis of the choroidal changes in patients with PPH. We also aim to investigate whether choroidal parameters could serve as noninvasive potential biomarkers reflecting the cardiological status in patients with PPH.

## Results

### Baseline characteristics

The study included 29 individuals with PPH and 37 healthy individuals constituting a control group. No statistically significant differences have been found between the groups in terms of age, sex, nicotine consumption, or systolic blood pressure. Additionally, no differences have been observed between the eyes of the participants from both groups as far as AL, IOP, or BCVA are concerned. Differences have been found in terms of diastolic blood pressure, and consequently, mean arterial pressure between the participants in the PPH group and the control group (in both cases, pressure was lower in the PPH group, *p* < 0.005); and caffeine consumption was higher in the control group (*p* = 0.017).

The group characteristics are presented in Table 1.

**Table 1:** Demographic and clinical characteristics of participants.

Variable	Total	PPH group	Control group	*p*
Number of patients	66	29	37	
Age [years], mean ± SD	52.62 ± 12.64	55.38 ± 15.40	50.46 ± 9.65	0.140
Sex. n (%)				
Female	34 (51.5)	13 (44.8)	21 (56.8)	0.475
Male	32 (48.5)	16 (55.2)	16 (43.2)	
BP Systolic [mmHg], mean ± SD	122.58 ± 17.41	118.41 ± 11.87	126.48 ± 20.78	0.069
BP Diastolic [mmHg], mean ± SD	77.53 ± 10.64	73.41 ± 9.24	81.39 ± 10.55	**0.003**
MAP [mmHg], mean ± SD	92.55 ± 11.31	88.41 ± 8.75	96.42 ± 12.17	**0.005**
Caffeine, n (%)	37 (56.1)	11 (37.9)	26 (70.3)	**0.017**
Nicotine, n (%)	10 (15.2)	4 (13.8)	6 (16.2)	> 0.999^1^
AL [mm], mean ± SD	23.45 ± 1.09	23.60 ± 1.19	23.33 ± 1.01	0.345
logMAR, median (Q1;Q3)	0.00 (0.00;0.00)	0.00 (0.00;0.00)	0.00 (0.00;0.00)	0.250²
IOP [mmHg], mean ± SD	15.49 ± 2.89	15.51 ± 3.76	15.47 ± 2.01	0.964
Spherical equivalent [D]	0.22 ± 1.33	0.12 ± 1.11	0.30 ± 1.50	0.590
K1 [D]	42.88 ± 1.47	42.83 ± 1.41	42.88 ± 1.53	0.804
K2 [D]	43.77 ± 1.53	43.83 ± 1.42	43.72 ± 1.64	0.725

AL, axial length; BP, blood pressure; IOP, intraocular pressure; K, corneal curvature; MAP, mean arterial pressure; PPH, precapillary pulmonary hypertension; Q1, quartile 1; Q3, quartile 3; D, diopter.

The groups compared by means of the chi-square test or Fisher exact test¹ for nominal variables and with t-test or Mann-Whitney U test² for continuous variables.

*p* < 0.05 in bold.

The cardiological characteristics of the PPH patients determined by history, physical examination, echocardiography, right heart catheterization, laboratory and functional tests as well as medication taken and the cause of PPH are reported in Table 2.

**Table 2:** Cardiological clinical characteristics of PPH group.

Variable	Value
PPH duration [years], median (Q1;Q3)	5.00 (3.00;13.00)
NT-proBNP [pg/ml], median (Q1;Q3)	227.55 (155.93;992.33)
Presence of peripheral edema, n (%)	4 (13.8)
6MWT [m], mean ± SD	437.38 ± 146.82
Echocardiographical parameters	
TAPSE [mm], mean ± SD	17.32 ± 5.68
Tricuspid regurgitation peak gradient [mmHg], mean ± SD	53.22 ± 25.57
eMPAP [mmHg], mean ± SD	39.04 ± 16.00
RAP [mmHg], mean ± SD	5.90 ± 4.17
RAA [cm^2^], median (Q1;Q3)	20.00 (17.00;27.00)
Right ventricle diameter in the 4CH view [cm], median (Q1;Q3)	4.60 (3.85;5.00)
Systolic pulmonary artery pressure [mmHg], mean ± SD	52.36 ± 26.71
Pharmacotherapy	
Sildenafil, yes, n (%)	16 (55.2)
Bosentan, yes, n (%)	11 (37.9)
Furosemid, yes, n (%)	11 (37.9)
PPH etiology	
Idiopathic, yes, n (%)	16 (55.2)
Surgical defect, yes, n (%)	1 (3.4)
Non-surgical defect, yes, n (%)	2 (6.9)
Connective tissue diseases, n (%)	1 (3.4)
Congenital, n (%)	8 (27.6)
CTEPH, n (%)	3 (10.3)

CTEPH, chronic thromboembolic pulmonary hypertension; eMPAP, estimated mean pulmonary arterial pressure; NT-proBNP, N-terminal pro-B-type Natriuretic Peptide; PPH, precapillary pulmonary hypertension; RAA, right atrial area; RAP, right atrial pressure; TAPSE, Tricuspid Annular Plane Systolic Excursion; 4CH, four-chamber; 6mWT, six minutes walking test; Q1, quartile 1; Q3, quartile 3; *p* < 0.05 in bold.

### Comparison of choroidal parameters in the PPH and control groups

Choroidal thickness (CT) and choroidal volume (CV) were significantly lower in the PPH group than in controls across all ETDRS subfields, and SFCT was also significantly lower (all BH-adjusted *p* ≤ 0.005). Other choroidal metrics (mTCA, mLA, mSA, mCVI) showed no significant between-group differences after BH correction (all BH-adjusted *p* > 0.05) (Table 3).

**Table 3:** Association between group (PPH vs. control) and choroidal parameters in multivariable regression models adjusted for blood pressure measures and caffeine intake.

Dependent variable:	Total	PPH Group	Control Group	β	95% CI	B	*p*	*R* ^2^	adjusted *R*^2^	B-H corr
Choroidal thickness [µm]:										
Outer T	247.26 ± 76.41	215.54 ± 80.23	271.27 ± 64.63	-66.840	-109.906; -23.775	-0.875	**0.003**	0.195	0.136	**0.003**
Inner T	271.22 ± 92.66	230.75 ± 97.83	301.84 ± 76.47	-88.580	-141.113; -36.041	-0.956	**0.001**	0.209	0.151	**0.001**
Central macular	284.26 ± 102.26	234.50 ± 103.98	321.92 ± 84.24	-105.198	-162.615; -47.781	-1.029	**0.001**	0.238	0.182	**0.001**
Outer N	225.48 ± 75.38	193.64 ± 74.29	249.57 ± 67.66	-66.890	-109.318; -24.462	-0.887	**0.003**	0.211	0.152	**0.003**
Inner N	267.34 ± 97.47	214.50 ± 89.92	308.44 ± 83.02	-107.700	-160.320; -55.080	-1.105	**< 0.001**	0.277	0.224	**< 0.001**
Outer S	287.25 ± 90.41	250.54 ± 98.24	315.03 ± 73.83	-77.224	-129.456; -24.993	-0.854	**0.005**	0.192	0.132	**0.005**
Inner S	290.74 ± 98.58	240.46 ± 96.70	328.78 ± 82.52	-106.864	-161.005; -52.724	-1.084	**< 0.001**	0.275	0.221	**< 0.001**
Outer I	251.15 ± 84.33	215.36 ± 86.84	278.24 ± 72.36	-85.593	-131.443; -39.743	-1.015	**< 0.001**	0.218	0.160	**< 0.001**
Inner I	270.31 ± 97.97	226.00 ± 101.61	303.84 ± 81.48	-95.531	-149.502; -41.561	-0.975	**0.001**	0.204	0.145	**0.001**
SFCT	285.26 ± 109.82	231.93 ± 107.65	325.62 ± 94.15	-113.963	-176.122; -51.804	-1.038	**0.001**	0.233	0.176	**0.001**
Choroidal Volume [mm^3^]:										
Outer T	1.30 ± 0.40	1.13 ± 0.43	1.43 ± 0.33	-0.351	-0.577; -0.124	-0.875	**0.003**	0.186	0.126	**0.003**
Inner T	0.42 ± 0.15	0.36 ± 0.15	0.47 ± 0.12	-0.142	-0.225; -0.059	-0.973	**0.001**	0.215	0.157	**0.001**
Central macular	0.22 ± 0.08	0.18 ± 0.08	0.25 ± 0.07	-0.083	-0.128; -0.037	-1.013	**0.001**	0.231	0.174	**0.001**
Outer N	1.17 ± 0.38	1.00 ± 0.35	1.30 ± 0.36	-0.380	-0.588; -0.172	-0.989	**0.001**	0.244	0.188	**0.001**
Inner N	0.42 ± 0.15	0.34 ± 0.15	0.48 ± 0.13	-0.168	-0.253; -0.083	-1.084	**< 0.001**	0.257	0.202	**< 0.001**
Outer S	1.50 ± 0.47	1.26 ± 0.47	1.68 ± 0.39	-0.499	-0.759; -0.238	-1.053	**< 0.001**	0.265	0.211	**< 0.001**
Inner S	0.45 ± 0.16	0.37 ± 0.16	0.52 ± 0.13	-0.175	-0.262; -0.088	-1.106	**< 0.001**	0.275	0.222	**< 0.001**
Outer I	1.32 ± 0.44	1.14 ± 0.46	1.45 ± 0.38	-0.450	-0.694; -0.206	-1.020	**0.001**	0.214	0.156	**0.001**
Inner I	0.42 ± 0.15	0.36 ± 0.16	0.48 ± 0.13	-0.148	-0.233; -0.064	-0.964	**0.001**	0.202	0.142	**0.001**
Total	7.29 ± 2.23	6.31 ± 2.36	8.03 ± 1.83	-2.144	-3.388; -0.900	-0.963	**0.001**	0.212	0.154	**0.001**
Other choroidal parameters:										
mTCA [µm^2^]	342 477.25 ± 102 933.08	316 721.88 ± 111 974.98	359 879.53 ± 93 926.44	-67276.600	-130522.999; -4030.265	-0.654	**0.038**	0.091	0.019	0.076
mLA [µm^2^]	225 238.37 ± 66 151.16	206 904.02 ± 71 612.61	237 626.45 ± 60 039.77	-46354.500	-86356.571; -6352.524	-0.701	**0.024**	0.103	0.032	0.076
mSA [µm^2^]	117 238.88 ± 38 489.53	109 817.86 ± 41 602.39	122 253.08 ± 35 948.58	-20922.100	-45143.792; 3299.623	-0.544	0.089	0.066	0.008	0.119
mCVI [%]	65.90 ± 2.80	65.49 ± 2.79	66.17 ± 2.81	-0.488	-2.100; 1.125	-0.187	0.546	0.023	0.055	0.546

β beta coefficient, B standardized beta, B-H corr Benjamini-Hochberg correction for multiple comparisons, CI confidence interval, CVI choroidal vascularity index, I inferior, LA luminal area, m macular, MAP mean arterial pressure, N nasal, PPH precapillary pulmonary hypertension, R² coefficient of determination, S superior, SA stromal area, SFCT subfoveal choroidal thickness, T temporal, TCA total choroidal area, *p* < 0.05 in bold. The conventional ETDRS grid with nine subfields, central macular field (central field within a 500-µm radius), four inner subfields (within a 500–1500-µm radius) and four outer subfields (within a 1500–3000-µm radius). Each row represents one multivariable model with group (PPH vs. control) as the independent variable and one choroidal parameter as the dependent variable. The confounders included in each model are systolic blood pressure, diastolic blood pressure, MAP, and caffeine intake. Benjamini–Hochberg (BH) correction for multiple comparisons was applied separately to choroidal thickness (10 comparisons), choroidal volume (10 comparisons), and other choroidal parameters (4 comparisons).

### Cut-off point calculation of the choroidal thickness and volume

**Fig. 1:**
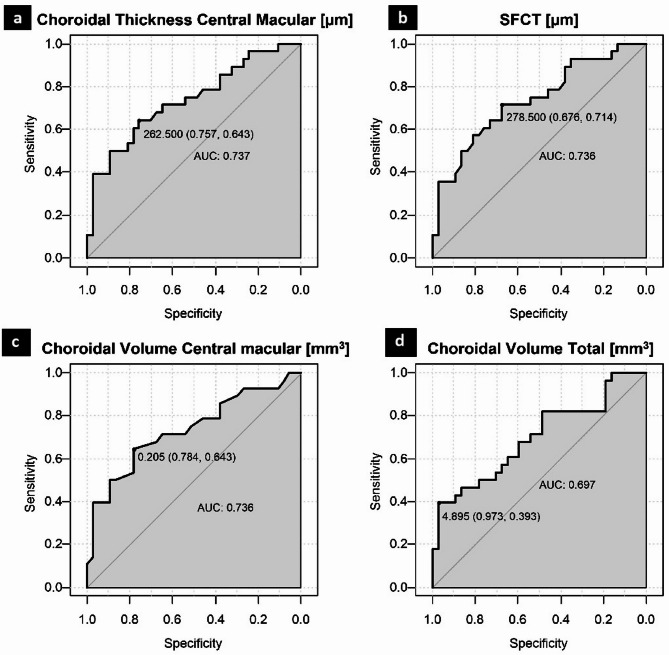
ROC (Receiver Operating Characteristic) curves of the selected choroidal parameters (a, b thickness; c, d volume) as discriminators between the PPH and control subjects. AUC, area under curve; PPH, precapillary pulmonary hypertension; SFCT, subfoveal choroidal thickness.

Receiver Operating Characteristic (ROC) curves were generated to determine the optimal cut-off points for the selected parameters to discriminate between the PPH patients and control subjects. The cut-off point calculation was based on Youden’s index. The sensitivity, specificity, accuracy, negative predictive value (NPV) and positive predictive value (PPV) of the received cut-off points were calculated as well (Fig. 1; Table 4).

**Table 4:** Performance of selected choroidal parameters in the prediction of precapillary pulmonary hypertension.

	PPH vs. Controls						
	Optimal cut-off point	AUC (95% CI)	Sensitivity (%)	Specificity (%)	Accuracy (%)	PPV (%)	NPV (%)
Choroidal Thickness Central macular [µm]	262.50	0.737 (0.612; 0.863)	64.3	75.7	70.8	66.7	73.7
SFCT [µm]	278.50	0.736 (0.611; 0.860)	71.4	67.6	69.2	62.5	75.8
Choroidal Volume Central macular [mm^3^]	0.205	0.736 (0.608; 0.863)	64.3	78.4	72.3	69.2	74.4
Choroidal Volume Total [mm^3^]	4.895	0.697 (0.564; 0.830)	39.3	97.3	72.3	91.7	67.9

AUC, area under curve; PPH precapillary pulmonary hypertension; PPV, positive predictive value; NPV, negative predictive value, SFCT, subfoveal choroidal thickness.

## Identification of parameters correlated with the choroidal thickness and volume in the PPH and control group

Keeping in mind that there is a significant difference in the CT and CV between the PPH patients and controls we have sought to identify the related clinical parameters. The univariable regression analyses of the association between the choroidal parameters (central macular choroidal thickness and volume), and clinical, demographic and ocular features are presented in Table 5 (PPH group) and Table 6 (control group). No significant associations have been found between choroidal parameters and age, sex, AL, nicotine use, MAP, disease duration, the WHO functional class, or medications that may affect choroidal parameters in PPH (sildenafil, bosentan), or other cardiological parameters, except for the right ventricle parameters in the four-chamber view [cm] (*p* < 0.05). In the control group, no associations have been found between clinical parameters and choroidal parameters.

**Table 5:** The univariable regression analysis of the factors associated with choroidal parameters in the PPH group.

Independent variable:	Central macular Choroidal Thickness [µm]						Central macular Choroidal Volume [mm^3^]					
	β	SE (Standard Error)	B	p	R2	adjusted R^2^	β	SE (Standard Error)	B	p	R2	Adjusted R^2^
Age [years]	-1.415	1.271	-0.209	0.276	0.045	0.009	-0.001	0.001	-0.195	0.311	0.039	0.002
Sex, male	-17.020	40.010	-	0.674	0.007	-0.031	-0.016	0.032	-	0.631	0.009	-0.029
MAP [mmHg]	-3.975	2.166	-0.335	0.078	0.115	0.081	-0.003	0.002	-0.312	0.101	0.100	0.066
PPH duration, [years]	4.092	2.856	0.301	0.166	0.089	0.046	0.004	0.002	0.323	0.138	0.102	0.059
Coffeine, yes	-18.360	41.640	-	0.663	0.007	-0.031	-0.017	0.033	-	0.616	0.009	-0.028
Nicotine, yes	-120.770	60.260	-	0.056	0.134	0.101	-0.094	0.049	-	0.065	0.125	0.091
IOP [mmHg]	-7.310	7.148	-0.197	0.316	0.039	0.002	-0.006	0.006	-0.198	0.313	0.039	0.002
AL [mm]	-11.810	16.990	-0.135	0.493	0.018	-0.019	-0.011	0.014	-0.155	0.431	0.024	-0.014
mCVI [%]	-4.726	9.511	-0.104	0.624	0.011	-0.034	-0.004	0.008	-0.099	0.638	0.010	-0.035
mTCA [µm^2^]	0.000	0.000	0.354	0.078	0.129	0.091	0.000	0.000	0.357	0.075	0.131	0.093
mLA [µm^2^]	0.001	0.000	0.374	0.062	0.144	0.107	0.000	0.000	0.376	0.059	0.146	0.109
mSA [µm^2^]	0.001	0.000	0.310	0.125	0.099	0.060	0.000	0.000	0.312	0.123	0.100	0.061
Central macular Choroidal Thickness [µm]							0.001	0.000	0.997	< **0.001**	0.994	0.994
Central macular Choroidal Volume [mm^3^]	1238.483	18.247	0.997	**< 0.001**	0.994	0.994						
WHO functional class 1–4	-19.970	37.440	-0.109	0.599	0.012	-0.029	-0.014	0.030	-0.093	0.655	0.008	-0.033
NT-proBNP [pg/ml]	-0.051	0.050	-0.214	0.318	0.048	0.002	0.000	0.000	-0.208	0.334	0.045	-0.001
Presence of peripheral edema, yes	62.200	58.170	-	0.297	0.049	0.006	0.049	0.047	-	0.313	0.046	0.003
6mWT [m]	0.037	0.147	0.052	0.804	0.003	-0.041	0.000	0.000	0.047	0.823	0.002	-0.041
TAPSE [mm]	1.710	3.904	0.093	0.666	0.008	-0.035	0.002	0.003	0.108	0.619	0.011	-0.032
Tricuspid regurgitation peak gradient [mmHg]	-0.105	0.892	-0.026	0.907	0.001	-0.049	0.000	0.001	-0.029	0.898	0.001	-0.049
eMPAP [mmHg]	-0.067	1.453	-0.010	0.964	0.001	-0.048	0.000	0.001	-0.018	0.935	0.001	-0.047
RAP [mmHg]	1.715	5.790	0.069	0.770	0.005	-0.050	0.001	0.005	0.060	0.802	0.004	-0.052
RAA [cm^2^]	-5.038	4.198	-0.267	0.243	0.064	0.019	-0.004	0.003	-0.270	0.238	0.066	0.021
Right ventricle 4CH view [cm]	-62.270	22.120	-0.485	**0.010**	0.248	0.217	-0.051	0.018	-0.494	**0.009**	0.253	0.222
Systolic pulmonary artery pressure [mmHg]	-0.998	0.864	-0.256	0.262	0.066	0.016	-0.001	0.001	-0.253	0.270	0.064	0.014
Sildenafil, yes	43.650	41.630	-	0.305	0.044	0.004	0.036	0.033	-	0.286	0.047	0.008
Bosentan, yes	58.650	39.330	-	0.150	0.088	0.049	0.048	0.032	-	0.147	0.089	0.049
Furosemid, yes	-14.640	41.870	-	0.730	0.005	-0.035	-0.013	0.034	-	0.702	0.006	-0.034
Idiopathic, yes	-14.770	42.000	-	0.728	0.005	-0.035	-0.017	0.034	-	0.625	0.010	-0.029

AL axial length, CVI choroidal vascularity index, eMPAP estimated mean pulmonary arterial pressure, IOP intraocular pressure, LA luminal area, m macular, MAP mean arterial pressure, NT-proBNP, N-terminal pro-B-type Natriuretic Peptide, PPH precapillary pulmonary hypertension, RAA right atrial area, RAP right atrial pressure, SA stromal area, SFCT subfoveal choroidal thickness, TAPSE tricuspid annular plane systolic excursion, TCA total choroidal area, β beta coefficient, B standardized beta, 4CH four-chamber, 6mWT six minutes walking test, *p* < 0.05 in bold.

**Table 6:** The univariate regression analysis of the factors associated with choroidal parameters in the control group.

Independent variable:	Central macular choroidal thickness [µm]						Central macular choroidal volume [mm^3^]					
	β	SE (Standard Error)	B	p	R2	Adjusted R^2^	β	SE (Standard Error)	B	P	R2	Adjusted R^2^
Age [years]	-1.086	1.464	-0.124	0.463	0.015	-0.013	-0.001	0.001	-0.129	0.443	0.017	-0.011
Sex, male	-15.600	28.230	-	0.584	0.009	-0.019	-0.010	0.022	-	0.652	0.006	-0.023
MAP [mmHg]	-0.584	1.236	-0.084	0.640	0.008	-0.027	0.000	0.001	-0.068	0.706	0.005	-0.029
Coffeine, yes	10.750	30.680	-	0.728	0.004	-0.025	0.009	0.024	-	0.727	0.004	-0.025
Nicotine, yes	23.970	37.890	-	0.531	0.011	-0.017	0.018	0.029	-	0.550	0.010	-0.018
IOP [mmHg]	2.692	5.140	0.088	0.604	0.008	-0.021	0.002	0.004	0.079	0.641	0.006	-0.022
AL [mm]	-21.160	13.580	-0.255	0.128	0.065	0.038	-0.016	0.010	-0.240	0.153	0.058	0.031
mCVI [%]	-4.592	5.000	-0.153	0.365	0.024	-0.004	-0.004	0.004	-0.168	0.321	0.028	0.000
mTCA [µm^2^]	0.001	0.000	0.859	**< 0.001**	0.739	0.732	0.000	0.000	0.857	**< 0.001**	0.734	0.727
mLA [µm^2^]	0.001	0.000	0.853	**< 0.001**	0.727	0.719	0.000	0.000	0.847	**< 0.001**	0.718	0.710
mSA [µm^2^]	0.002	0.000	0.822	**< 0.001**	0.676	0.667	0.000	0.000	0.824	**< 0.001**	0.679	0.669
Central macular Choroidal Thickness [µm]							0.001	0.000	0.998	**< 0.001**	0.995	0.995
Central macular Choroidal Volume [mm^3^]	1266.120	14.919	0.998	**< 0.001**	0.995	0.995						

AL axial length, CVI choroidal vascularity index, IOP intraocular pressure, LA luminal area, m macular, MAP mean arterial pressure, SA stromal area, TCA total choroidal area, β beta coefficient, B standardized beta, 6mWT six minutes walking test, *p* < 0.05 in bold.

As choroidal thickness and volume are correlated, those associations have been detected through the univariate regression analysis in both groups. On the other hand, the TCA is correlated with choroidal thickness. However, it has been measured on a single scan and consists of LA and SA. Since those parameters are correlated, the associations among them have been detected through the univariate regression analyses in the control group but not in the PPH group. Those findings should be interpreted with caution due to the low power of the CVI calculation.

## Discussion

In our cohort we are able to show the decreased choroidal thickness and volume in all the ETDRS subfields in the patients with PPH as compared to the controls. The number of the included patients does not allowed to draw definitive conclusions regarding the CVI values. In the PPH group, the choroidal thickness and volume are not correlated with the cardiological parameters, except for the right ventricle in a four-chamber (4CH) view [cm].

The data regarding the choroidal thickness and volume in patients with PH are inconclusive and the patients’ groups differ significantly. Gu et al. observed a greater subfoveal choroidal thickness in individuals with primary PH as compared to healthy individuals^18^. However, the study group consisted solely of individuals with idiopathic pulmonary hypertension whereas in our study, the patients from this group have made up 57% (43% belonged to other categories). Martiano et al. also noted a thickened choroid in a patient with idiopathic pulmonary hypertension treated with tadalafil. They speculated that phosphodiesterase 5 inhibitors (PDE-5), such as tadalafil and sildenafil, by affecting vasodilation, may have increased choroidal blood flow and choroidal thickness^19^. On the other hand, in a study involving patients with chronic thromboembolic pulmonary hypertension, several microvascular changes were observed in the retina and choroid, including a reduction in mean subfoveal choroidal thickness, as compared to individuals in the control group^20^. That corresponds with our results, although individuals from that group have made up only 10.3% (3 individuals) in our study. We assume that an acute or short-lasting increased pressure in the superior vena cava transmitted to the ophthalmic veins and choroidal circulation could result in an increased choroidal thickness. Albeit, long-lasting one with concomitant hypoxia could lead to the choroidal luminal and stromal remodeling with resultant decrease in its thickness. Such a theory would support our results.

As we have found significant differences in the choroidal thickness and volume, the ROC curves are used for distinguishing between the PPH group and the controls. The AUC values may suggest the potential utility of those parameters, likely in conjunction with the additional ones (whether OCT-based or not).

The interpretation of our results must acknowledge the vasoactive medication taken by the patients with PPH taking into account not only their mechanisms of action but potential adverse drug reactions as well. Our study has included 16 patients taking sildenafil at a daily dose ranging from 2 × 25 mg up to 3 × 40 mg and 11 patients taking bosentan (2 × 125 mg).

Potential effects of sildenafil on ocular circulation have been the focus of several studies. A significant increase in ocular blood flow has been reported^21,22^. Kim et al. have reported that both the choroidal perfusion and thickness increase in response to systemic sildenafil^23^. However, in a study where healthy volunteers took 200 mg of sildenafil, small, statistically insignificant changes in choroidal thickness were observed. However, measurements were performed using ultrasonography, which may have resulted in less accurate results as compared to the OCT imaging^24^. In another study using EDI-OCT, a significant increase in choroidal thickness was observed in healthy individuals 1 and 3 h after having taken 100 mg of sildenafil^25^. There are cases describing even central serous chorioretinopathy (CSC) associated with the use of sildenafil^26,27^. In summary, a number of studies have examined the effects of PDE5 inhibitors on ocular blood flow in healthy volunteers, men with erectile dysfunction, age-related macular degeneration or pre-existing eye disorders, using a variety of techniques and with varying results. Effects have been minor and inconsistent; inconsistencies in the reported changes may reflect the difficulty in measuring relatively small changes^28^.

Other medications used in the treatment of PPH may also induce specific changes in ocular microcirculation, such as bosentan, which causes dilation of the retinal vessels and increased blood flow in the choroid and retina^29^.

Our results do not allow to draw conclusions regarding the CVI values, as the study population was too small to provide adequate power for this parameter. There is no data in the related literature we could refer to. Based on our results we may only speculate that both luminal and stromal areas decrease and in consequence the CVI remains unchanged. That assumption requires to be verified in further research.

We have assumed common pathomechanisms within choroidal and other vessels in the PPH patients. However, with minor exceptions, we have not found any associations of the choroidal thickness and volume with cardiological parameters. We speculate that the complex and multifactorial pathomechanisms within choroidal vessels (including their backup capacity, endothelium alterations, hypoxia, and vasoactive medication taken) as well as the medications taken and the PPH etiological diversity might prevent a direct correlation. However, future studies might further explore the suitability of choroidal parameters for the assessment of the cardiological status of PPH patients.

It remains to be elucidated how altered choroidal parameters, as found also in our study, affect the choroidal and retinal function. It is noteworthy that retina is nourished by blood from two independent sources. The inner retina is supplied by the central retinal artery, the outer retina is perfused mainly by the choroid^30,31^. Choroidal perfusion impairment is important especially in the foveal region, where the retinal vascular supply is lacking (foveal avascular zone). The choroid is a highly vascularized tissue in the body, which is responsible for over 70%-90% of the blood flow in the eye at a high flow rate to ensure the photoreceptors high metabolic demand^32,33^. Choroidal vasculature has the end-arterial character which makes this layer prone to inflammation and ischemia in various multisystemic diseases including systemic sclerosis^34^. All in all, the anatomical-functional relationship as well as causal one, requires further studies.

In terms of limitations of this study, the study group is relatively small and composed of PPH patients of various etiologies, limiting the power of our calculations. However, pulmonary hypertension is a rare disease, affecting approximately 15–50 individuals per million in the USA and Europe^35^. Additionally, some limitations arose from the fact that the study was conducted during the COVID-19 pandemic in Poland and worldwide. Due to the study being conducted at a single center, generalizability of our results may be limited. Our conclusions are relevant to the described and probably similar cohorts but would need to be validated in different PPH cohorts. In addition, the low number of PPH participants has prevented the subgroup analyses based on the medication taken or the category of PPH, which, despite having common pathological features, varies in clinical presentation, mechanisms, and etiology. For the sake of a larger group, we have included the patients with PPH of various etiology.

Given the strengths of our study, to the best of our knowledge, this study is the first to analyze the choroidal thickness and volume in a 6 mm macular area in patients with PPH. It is also the first attempt to verify potential suitability of choroidal parameters as biomarkers of cardiological status in patients with PPH. Additionally, the parameters we have analyzed, such as the CVI and choroidal volume, are relatively rarely assessed in studies, yet they provide additional detailed data describing the choroid. Furthermore, in order to avoid physiological changes in choroidal properties throughout the day, the OCT scans were performed at a similar time.

Future studies may include more patients from various clinical settings facilitating the subgroup analyses based on etiology, medication taken, etc. Longitudinal studies would reflect choroidal changes over time. It would be advisable to use both the OCT and OCT-A to gain a better insight into the occurring changes and their pathophysiology and obtain complementary results based on the enface and transverse OCT scans. Adding ultrasound of the retrobulbar vessels and internal carotid artery would broaden our knowledge of blood supply to the eyeball in patients with PPH.

## Conclusions

Our study provides insights into the pathophysiology of the choroid in patients with PPH. In our cohort the presence of PPH is associated with the decreased macular choroidal thickness and volume. Albeit, in general, those choroidal parameters do not correspond to most of the cardiovascular phenotype characteristics, so they do not seem to be suitable as biomarkers for the PH evaluation. As our results do not give a definitive answer to that issue, further studies are needed to establish a potential role and meaning of the CVI values in patients with PPH.

## Materials and methods

This prospective, single-center, cross-sectional study was conducted between 2021 and 2023 at the Ophthalmology Department of the Medical University of Bialystok. The study involved 29 adult PPH patients admitted for routine clinical assessment to the Department of Cardiology and Internal Diseases of the Medical University of Bialystok, that were on stable PPH pharmacotherapy.

The control group was composed of 37 healthy adult patients (self-reported) undergoing routine ophthalmological assessments. One randomly selected eye per patient was included in the analyses.

The protocol of the study was approved by the local Bioethics Committee at the Medical University of Bialystok (decision no APK.002.290.2020), and the study was conducted in accordance with the Declaration of Helsinki. The informed written consent was obtained from each patient in both groups.

The diagnostic ophthalmologic workup for all participants included refraction, best corrected visual acuity (BCVA) in Snellen converted to logMAR, intraocular pressure (IOP) measured with a Pascal dynamic contour tonometer (DCT; Zeimer Ophthalmic Systems AG, Port, Switzerland), slit-lamp biomicroscopy, keratometry and AL measured with a Tomey OA-2000 biometer (Nagoya, Japan), spherical equivalent with Topcon KR-8900 auto kerato-refractometer (Tokyo, Japan), dilated fundus examination, and enhanced depth imaging optical coherence tomography (EDI-OCT; Heidelberg Engineering GmbH, Heidelberg, Germany; 2016). Blood pressure was measured in the sitting position after 5 min of rest, and OCT images were obtained immediately afterwards for all the patients. Data regarding age, sex, current smoking status, PPH duration, and details of systemic treatment were recorded. The PPH patients were diagnosed according to the European Society of Cardiology guidelines with right heart catheterization, that confirmed precapillary pulmonary hypertension (estimated mean pulmonary artery pressure [eMPAP] ≥ 25 mmHg and pulmonary artery wedge pressure [PAWP] ≤ 15 mmHg) with elevated pulmonary vascular resistance (PVR) (> 3 Wood units [WU])^1^. PPH group included patients with idiopathic pulmonary arterial hypertension as well as related to congenital heart defects or associated to connective tissue diseases, and patients with chronic thromboembolic pulmonary hypertension. Patients with significant lung disease or PH secondary to left heart diseases were not included in the study. The following parameters characterizing PPH were obtained: disease history, physical examination, echocardiography, right heart catheterization, laboratory and functional tests: WHO functional class, presence of peripheral edema, N-terminal pro B-type Natriuretic Peptide concentration and results of 6-minute walk tests. In order to ensure proper classification, echocardiographic parameters like tricuspid annular plane systolic excursion (TAPSE), right ventricular systolic function, tricuspid regurgitation peak gradient, estimated mean pulmonary arterial pressure (eMPAP), estimated pulmonary artery systolic pressure, right atrial pressure (RAP), right atrial area (RAA), right ventricular diameter in four-chamber (4CH) view were included. PPH treatment strategies were carefully analyzed^36^. Exclusion criteria applicable to both groups encompassed ophthalmological factors: the presence of fundus pathology, ametropia ≥ 3 diopters, cataract surgery shorter than 12 months prior to examination, history of posterior segment surgery, retinal laser treatment, and poor quality of OCT scans (< 25 dB).

### OCT imaging and analysis

**Fig. 2:**
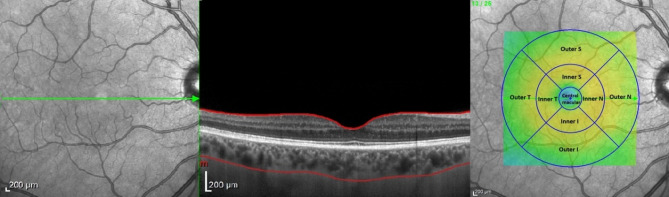
Analyzed choroidal and retinal regions superimposed on the fundus image.

The OCT imaging protocol for macula (Fig. 2) was composed of 25 raster scans (20°× 20°) and a linear 30° B-scan centered at the fovea. Choroidal thickness and volume were determined in the same manner as described in detail in our previous studies^34,37^. Briefly, the internal limiting membrane (ILM) and Bruch’s membrane (BM) were detected automatically, while the choroidal–scleral junction (CSJ) was manually marked on each scan. Retinal parameters were calculated from the ILM to the BM, and choroidal parameters - from the BM to the CSJ. Average thickness and volume maps were created automatically according to the conventional ETDRS grid with nine subfields including the central macular subfield (central field within a 500 µm radius)^38^. Values of the choroidal parameters were calculated by subtracting retinal parameters from the summed retinal and choroidal parameters. The subfoveal choroidal thickness (SFCT) was defined as the distance between the BM and the CSJ at the fovea and was measured automatically. In our study, we used the term ‘subfoveal’ to specifically refer to the central point directly beneath the fovea (i.e., a single-point measurement), whereas ‘central macular’ referred to the 500 μm radius region centered on the fovea.

**Fig. 3:**
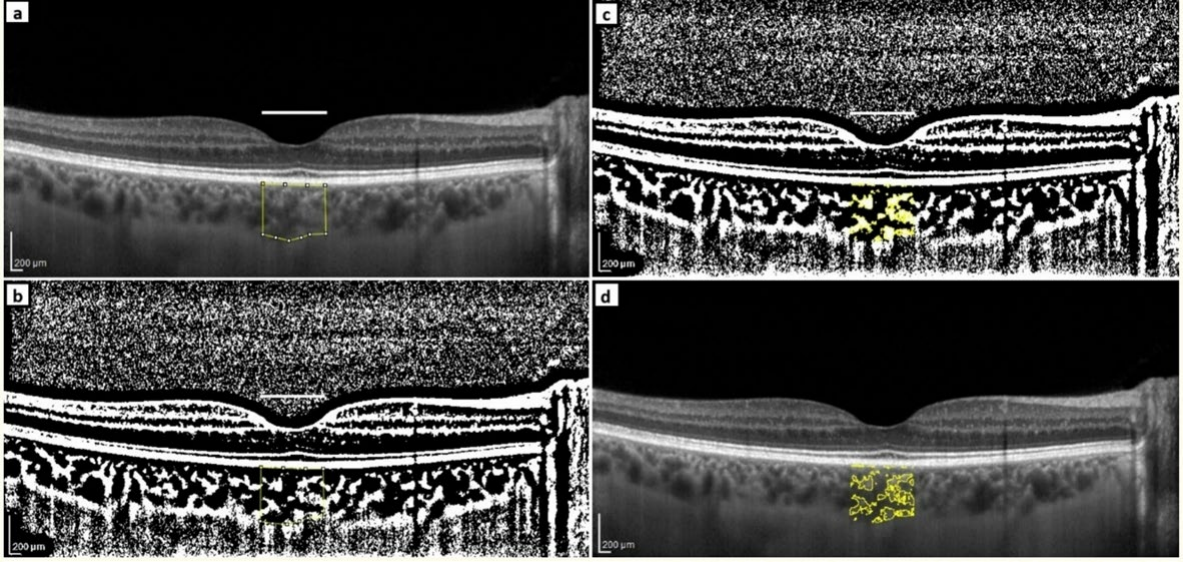
Image binarization of the choroid. **a** Total choroidal area marked on the analyzed enhanced depth imaging optical coherence tomography (EDI-OCT) scan. The horizontal white line length equals 1000 μm. **b** The Niblack auto local threshold tool applied. **c** The color threshold tool applied highlighting the luminal area. **d** Overlay of luminal area on the original OCT scan.

CVI assessment was performed according to the protocols of Sonoda et al.^39^ and Agrawal et al.^13^, with minor modifications as detailed in a step-by-step description with screenshots in our previous publication^34^. Binarization of the macular choroidal area was performed by two researchers (AZ and BP) (Fig. 3). The macular region was scanned using a single horizontal line scan (30°) centered on the fovea, with 100 frames averaged in a B-scan. The measurement area was defined as 1000 μm in width and centered on the fovea. The total choroidal area (TCA) was selected from the outer boundary of the RPE–BM layer to the CSJ using the polygon selection tool. The images were converted into 8-bit images to allow for the application of the Niblack auto local threshold tool. The binarized images were reconverted into RGB images to allow for the color threshold tool to be used for selecting dark pixels that represented vascularized areas. The luminal area (LA) and TCA were measured, while the stromal area (SA) was calculated by subtracting the LA from the TCA. The CVI was determined as the ratio of the LA to the TCA (%). The parameters were assessed by two experienced researchers (AZ and BP) at the same time of the day (8–10 am) in both groups, with the averages from 2 measurements included in the analysis. Any doubts were resolved through discussion. The repeatability of measurements between observers was determined using the intraclass correlation coefficient (ICC) and overall agreement. The ICC was high, as shown in Table 7.

**Table 7:** Intraclass correlation coefficient of choroidal parameters measurements between two raters in two groups.

	PPH Group:		Control group:	
	ICC	95% CI	ICC	95% CI
mTCA [µm^2^]	0.983	0.968–0.991	0.966	0.944–0.979
mLA [µm^2^]	0.986	0.974–0.992	0.968	0.946–0.981
mSA [µm^2^]	0.976	0.953–0.987	0.959	0.935–0.974
mCVI [%]	0.936	0.891–0.963	0.948	0.919–0.967

CI, confidence interval; CVI, choroidal vascularity index; ICC, Intraclass correlation coefficient assuming the two-way model and absolute agreement; LA, luminal area; m, macular; PPH precapillary pulmonary hypertension; SA, stromal area; TCA, total choroidal area.

According to the related literature^40^, the ICC values below 0.5 are indicative of poor reliability, the values between 0.5 and 0.75 indicate moderate reliability, the values between 0.75 and 0.9 indicate good reliability, and the values greater than 0.90 indicate excellent reliability.

With respect to ocular magnification correction, axial and transverse measurements must be handled separately. Axial depth on the Heidelberg Spectralis is optically calibrated for tissue refractive indices (e.g., choroidal thickness), so axial scaling is independent of ocular magnification and requires no further correction^41^. In contrast, the Spectralis does not auto-correct lateral scaling for individual axial lengths^42^. However, because our PPH and control groups did not differ in axial length, corneal curvature, or spherical equivalent (Table 1; all *p* > 0.05), any residual magnification error is likely to be minimal and unlikely to bias between-group comparisons. Moreover, our primary lateral metric, the CVI, is the ratio of LA to TCA ^13^, so a uniform scaling factor applies equally to numerator and denominator and thus has no effect on the CVI. Together, these factors render magnification error minimal and non-differential; therefore, we omitted individual ocular magnification corrections.

### Post-hoc power calculation

The relatively small number of patients is due to the rarity of PPH and the sample size was partly determined by the number of participants who had met our inclusion criteria.

The post-hoc power calculation, assuming α = 0.05 and using the means and SDs obtained in the study for the comparison of PPH and controls patients, resulted in a power (1-β) of 0.96 for central macular choroidal thickness, volume, and SFCT, and 0.90 for total choroidal volume. The power calculation for the CVI equaled 0.16.

### Statistical analysis

Data included one eye per patient, randomly selected (left/right) by an independent statistician, to avoid potential bias from selection of only right or only left eyes^43^. The choroidal assessments by two investigators were averaged. Data are presented as n (% of group) for nominal variables or as mean ± SD or median (Q1; Q3) for continuous variables, as appropriate. Normality of distribution was assessed by means of using Shapiro-Wilk test as well as skewness and curtosis values. The comparison of the groups was based on tests: Fisher exact test or chi-square test for nominal data, Welch t-test or Mann-Whitney U test for continuous data, as appropriate. Additionally, MD (mean difference) between both groups was calculated, including 95% confidence interval (CI). Predictive performance of the choroidal thickness for PPH vs. controls was compared using the area under the receiver operating characteristic curve (AUC). Sensitivity and specificity values were reported at the cutoff points determined by the Youden index. The univariable linear regression analysis was carried out to determine the relationship between choroidal and cardiological parameters. Additionally, a multivariable regression analysis was performed for the selected parameters, with group (PPH vs. control) as the independent variable and the following confounders: systolic blood pressure, diastolic blood pressure, MAP, and caffeine intake. Benjamini–Hochberg correction for multiple comparisons was applied to control the false discovery rate at 5%, and both unadjusted and BH-adjusted p-values were reported. Benjamini–Hochberg adjustment was performed using the p.adjust function in R. For each linear regression model, the coefficient of determination (R^2^) and adjusted coefficient of determination (adjusted R^2^) were calculated and reported as measures of model fit.

The statistical analysis was conducted by means of R 4.2.1. statistical software (R Core Team (2022). R: Language and environment for statistical computing by R Foundation for Statistical Computing, Vienna, Austria). The post hoc power calculation was carried out by means of G*Power 3.1.9.7. software. All calculations were based on α = 0.05.

## Data Availability

We agree to make data and materials supporting the results or analyses presented in our paper available upon reasonable request, unless we are unable to do so for ethical, privacy, or security concerns. Please address the corresponding author Diana Anna Dmuchowska at diana.dmuchowska@umb.edu.pl.

## References

[1] Humbert, M. et al. Pathology and pathobiology of pulmonary hypertension: state of the Art and research perspectives. *Eur. Respir J.*, **53**, 1 (2019). 10.1183/13993003.01887-2018 PMC6351340 30545970

[2] Galie, N. et al. 2015 ESC/ERS guidelines for the diagnosis and treatment of pulmonary hypertension. *Kardiol Pol.* **73** (12), 1127–1206 (2015). 26727670 10.5603/KP.2015.0242

[3] Bousseau, S. et al. Pathophysiology and new advances in pulmonary hypertension. *BMJ Med.* **2** (1), e000137 (2023). 37051026 10.1136/bmjmed-2022-000137 PMC10083754

[4] Hemnes, A. R. & Brittain, E. L. Autonomic nervous system in pulmonary arterial hypertension: time to rest and digest. *Circulation* **137** (9), 925–927 (2018). 29483171 10.1161/CIRCULATIONAHA.117.032355 PMC5830155

[5] Kazimierczyk, R. & Kamiński, K. The role of platelets in the development and progression of pulmonary arterial hypertension. *Adv. Med. Sci.* **63** (2), 312–316 (2018). 29885631 10.1016/j.advms.2018.04.013

[6] Faure, C. et al. Primary pulmonary arterial hypertension diagnosed via its ophthalmic features in an adult: diagnosis and therapeutic challenges. *Br. J. Ophthalmol.* **96** (3), 459–460 (2012). 22049487 10.1136/bjo.2010.187468

[7] Paire, V. et al. [Familial primary pulmonary hypertension revealed by the association of bilateral chemosis, subacute myopia, and exophthalmos]. *J. Fr. Ophtalmol*. **29** (10), e27 (2006). 17211315 10.1016/s0181-5512(06)73911-0

[8] Krohn, J. & Bjune, C. Uveal effusion and angle-closure glaucoma in primary pulmonary hypertension. *Am. J. Ophthalmol.* **135** (5), 705–706 (2003). 12719080 10.1016/s0002-9394(02)02090-1

[9] Lewczuk, N. et al. Ocular manifestations of pulmonary hypertension. *Surv. Ophthalmol.* **64** (5), 694–699 (2019). 30849428 10.1016/j.survophthal.2019.02.009

[10] Delaey, C. & Van De Voorde, J. Regulatory mechanisms in the retinal and choroidal circulation. *Ophthalmic Res.* **32** (6), 249–256 (2000). 11015035 10.1159/000055622

[11] Nickla, D. L. & Wallman, J. The multifunctional choroid. *Prog Retin Eye Res.* **29** (2), 144–168 (2010). 20044062 10.1016/j.preteyeres.2009.12.002 PMC2913695

[12] Ostrin, L. A. et al. New insights, challenges, and potential significance for human myopia. *Invest. Ophthalmol. Vis. Sci.* **64** (6), 4 (2023). 37126359 10.1167/iovs.64.6.4 PMC10153586

[13] Agrawal, R. et al. Choroidal vascularity index as a measure of vascular status of the choroid: measurements in healthy eyes from a population-based study. *Sci. Rep.* **6**, 21090 (2016). 26868048 10.1038/srep21090 PMC4751574

[14] Dmuchowska, D. A. et al. Quantitative assessment of choroidal parameters in patients with various types of diabetic macular oedema: A Single-Centre Cross-Sectional analysis. *Biology (Basel)* **10**, 8 (2021). 10.3390/biology10080725 PMC8389323 34439957

[15] Gupta, C. et al. Choroidal structural analysis in eyes with diabetic retinopathy and diabetic macular edema-A novel OCT based imaging biomarker. *PLoS One*. **13** (12), e0207435 (2018). 30533048 10.1371/journal.pone.0207435 PMC6289408

[16] Tan, K. A. et al. Choroidal vascularity index - a novel optical coherence tomography parameter for disease monitoring in diabetes mellitus? *Acta Ophthalmol.* **94** (7), e612–e616 (2016). 27151819 10.1111/aos.13044

[17] Kılınç Hekimsoy, H. et al. Analysis of retinal and choroidal microvasculature in systemic sclerosis: an optical coherence tomography angiography study. *Eye (Lond)*. **34** (4), 763–770 (2020). 31554941 10.1038/s41433-019-0591-z PMC7093531

[18] Gu, S. et al. Optical coherence tomography angiography findings of microvascular and neural changes in primary pulmonary hypertension. *Retina* **41** (4), 784–792 (2021). 32773605 10.1097/IAE.0000000000002940 PMC7989611

[19] Martiano, D. et al. Acute serous retinal detachment in idiopathic pulmonary arterial hypertension. *Retin Cases Brief. Rep.* **11** (3), 261–265 (2017). 27203562 10.1097/ICB.0000000000000336

[20] Krajewski, P. et al. Optical coherence tomography angiography findings in patients with chronic thromboembolic pulmonary hypertension. *Retina* **42** (12), 2354–2360 (2022). 36007170 10.1097/IAE.0000000000003607

[21] Paris, G. et al. Sildenafil increases ocular perfusion. *Int. Ophthalmol.* **23** (4–6), 355–358 (2001). 11944862 10.1023/a:1014410932321

[22] Polak, K. et al. Effects of sildenafil on retinal blood flow and flicker-induced retinal vasodilatation in healthy subjects. *Invest. Ophthalmol. Vis. Sci.* **44** (11), 4872–4876 (2003). 14578411 10.1167/iovs.03-0177

[23] Kim, D. Y. et al. Measurement of choroidal perfusion and thickness following systemic sildenafil (Viagra(^®^)). *Acta Ophthalmol.* **91** (2), 183–188 (2013). 22974308 10.1111/j.1755-3768.2011.02305.x PMC3528845

[24] McCulley, T. J. et al. Effects of sildenafil citrate (Viagra) on choroidal congestion. *Ophthalmologica* **216** (6), 455–458 (2002). 12566892 10.1159/000067549

[25] Vance, S. K., Imamura, Y. & Freund, K. B. The effects of sildenafil citrate on choroidal thickness as determined by enhanced depth imaging optical coherence tomography. *Retina* **31** (2), 332–335 (2011). 20975620 10.1097/IAE.0b013e3181eef0ae

[26] Quiram, P. et al. Viagra-associated serous macular detachment. *Graefes Arch. Clin. Exp. Ophthalmol.* **243** (4), 339–344 (2005). 15756578 10.1007/s00417-004-1099-0

[27] Aliferis, K. et al. Should central serous chorioretinopathy be added to the list of ocular side effects of phosphodiesterase 5 inhibitors? *Ophthalmologica* **227** (2), 85–89 (2012). 22156704 10.1159/000333824

[28] Dündar, S. O. et al. Effects of sildenafil on blue-on-yellow and white-on-white Humphrey perimetry in 3 months regular use. *Eye (Lond)*. **20** (7), 810–813 (2006). 16052253 10.1038/sj.eye.6702017

[29] Resch, H. et al. Effect of dual endothelin receptor Blockade on ocular blood flow in patients with glaucoma and healthy subjects. *Invest. Ophthalmol. Vis. Sci.* **50** (1), 358–363 (2009). 18719081 10.1167/iovs.08-2460

[30] Pournaras, C. J. et al. Regulation of retinal blood flow in health and disease. *Prog Retin Eye Res.* **27** (3), 284–330 (2008). 18448380 10.1016/j.preteyeres.2008.02.002

[31] Böhm, E. W. et al. Methods to measure blood flow and vascular reactivity in the retina. *Front. Med. (Lausanne)*. **9**, 1069449 (2022). 36714119 10.3389/fmed.2022.1069449 PMC9877427

[32] Paczwa, K. et al. Ocular manifestation in systemic Sclerosis-A literature review. *Life (Basel)*, **14**, 5 (2024). 10.3390/life14050627 PMC11122095 38792647

[33] Agrawal, R. et al. Exploring choroidal angioarchitecture in health and disease using choroidal vascularity index. *Prog Retin Eye Res.* **77**, 100829 (2020). 31927136 10.1016/j.preteyeres.2020.100829

[34] Pieklarz, B. et al. Macular choroidal thickness, volume, and vascularity index in patients with systemic sclerosis. *Graefes Arch. Clin. Exp. Ophthalmol.* **262** (5), 1475–1487 (2024). 38133798 10.1007/s00417-023-06342-4 PMC11031445

[35] Levine, D. J. Pulmonary arterial hypertension: updates in epidemiology and evaluation of patients. *Am. J. Manag Care*. **27** (3 Suppl), S35–S41 (2021). 33710842 10.37765/ajmc.2021.88609

[36] Humbert, M. et al. [2022 ESC/ERS guidelines for the diagnosis and treatment of pulmonary hypertension]. *G Ital. Cardiol. (Rome)*. **24** (4), 1e–116e (2023). 36995376 10.1714/4014.39906

[37] Sidorczuk, P. et al. Foveal avascular zone does not correspond to choroidal characteristics in patients with diabetic retinopathy: A Single-Center Cross-Sectional analysis. *Diabetes Metab. Syndr. Obes.* **14**, 2893–2903 (2021). 34234487 10.2147/DMSO.S318860 PMC8254029

[38] Grading diabetic retinopathy. From stereoscopic color fundus photographs–an extension of the modified airlie house classification. ETDRS report number 10. Early treatment diabetic retinopathy study research group. *Ophthalmology* **98** (5 Suppl), 786–806 (1991). 2062513

[39] Sonoda, S. et al. Luminal and stromal areas of choroid determined by binarization method of optical coherence tomographic images. *Am. J. Ophthalmol.* **159** (6), 1123–1131e1 (2015). 25790737 10.1016/j.ajo.2015.03.005

[40] Koo, T. K. & Li, M. Y. A guideline of selecting and reporting intraclass correlation coefficients for reliability research. *J. Chiropr. Med.* **15** (2), 155–163 (2016). 27330520 10.1016/j.jcm.2016.02.012 PMC4913118

[41] Salmon, A. E. et al. Axial scaling is independent of ocular magnification in OCT images. *Invest. Ophthalmol. Vis. Sci.* **59** (7), 3037–3040 (2018). 30025118 10.1167/iovs.17-23549 PMC6005622

[42] Bennett, A. G., Rudnicka, A. R. & Edgar, D. F. Improvements on littmann’s method of determining the size of retinal features by fundus photography. *Graefes Arch. Clin. Exp. Ophthalmol.* **232** (6), 361–367 (1994). 8082844 10.1007/BF00175988

[43] Armstrong, R. A. Statistical guidelines for the analysis of data obtained from one or both eyes. *Ophthalmic Physiol. Opt.* **33** (1), 7–14 (2013). 23252852 10.1111/opo.12009

